# Associations between Alcohol-Free Sources of Reinforcement and the Frequency of Alcohol and Cannabis Co-Use among College Freshmen

**DOI:** 10.3390/ijerph20042884

**Published:** 2023-02-07

**Authors:** Daiil Jun, Tera L. Fazzino

**Affiliations:** 1 Department of Psychology, University of Kansas, Lawrence, KS 66045, USA; 2 Cofrin Logan Center for Addiction Research and Treatment, University of Kansas, Lawrence, KS 66045, USA

**Keywords:** alcohol, behavioral economics, co-use, heavy drinking, marijuana, young adults

## Abstract

Co-use of alcohol and cannabis is common among young adults in the United States. A behavioral economics framework indicates that greater engagement in substance-free sources of reinforcement may be protective against co-use frequency. The current study tested the association between proportionate alcohol-free reinforcement and the frequency of co-use among college freshmen. Participants (N = 86) were freshmen who enrolled in a freshman orientation course and completed surveys at the beginning of the semester. Past month alcohol use, cannabis use, and reinforcement from alcohol-free and alcohol-involved activities were assessed. A zero-inflated Poisson regression was used to test the association between proportionate alcohol-free reinforcement and days of co-use. The results indicated that proportionate alcohol-free reinforcement was negatively associated with co-use days in the count model when controlling for alcohol use days and gender as covariates (β: −3.28, *p* = 0.016). Proportionate alcohol-free reinforcement did not significantly differentiate individuals who did not engage in co-use in the zero-inflated model (β: −1.68, *p* = 0.497). The study suggested that greater proportionate alcohol-free reinforcement may be associated with lower engagement in the co-use of alcohol and cannabis among young adults. Increasing engagement in alcohol-free sources of reinforcement may be considered a target for co-use prevention or harm reduction efforts.

## 1. Introduction

Alcohol and cannabis are two substances that young adults commonly use in the United States (US); according to a national survey of US college students, most reported alcohol use (62%), and approximately one fifth reported cannabis use (26%) [1]. Moreover, young adults often use both substances, most commonly in a pattern of simultaneous use [2,3,4], likely because cannabis can enhance the rewarding effects of alcohol [5,6]. The prevalence of co-use is high, with almost one-quarter of college students (24%) reporting co-use of alcohol and cannabis as of 2018 [7]. Furthermore, the prevalence of co-use has increased by 7% over the past 20 years [7] and has occurred against the backdrop of public policy changes in many US states that have legalized medicinal and/or recreational cannabis [8]. The increased prevalence of co-use and legislative changes have yielded concerns regarding the potential health consequences of alcohol and cannabis co-use [9,10]. In the literature, co-use of alcohol and cannabis has consistently been associated with greater negative consequences, including blackouts, driving under the influence, and social consequences, compared to use of either alcohol or cannabis alone [11,12]. Thus, young adults may experience more harmful consequences from alcohol and cannabis co-use, and research is needed to understand factors that may contribute to and protect against co-use among young adults.

Behavioral economics provides a framework to conceptualize alcohol use and may be extended to understand alcohol and cannabis co-use among young adults. According to behavioral economics, risky alcohol use may result from the interaction between strong valuation and frequent use of alcohol as a primary source of reinforcement [13,14]. Consequently, individuals may engage in fewer alternative activities that do not involve alcohol, a pattern that may further narrow a behavioral repertoire to reliance on alcohol-related activities for reinforcement. Overall, the existing literature strongly supports the premise that young adults who obtain greater reinforcement from alcohol-related activities engage in more frequent alcohol use [15,16,17,18,19]. Initially, in this area of the literature, the degree of reinforcement from alcohol-related (or alcohol-free) activities was examined as a summary score [20]. However, in closer alignment with behavioral economic framework, researchers have highlighted the importance of characterizing alcohol-related reinforcement as a proportion, to contextualize alcohol-related reinforcement relative to the total reinforcement individuals may experience [21,22]. Thus, most of the current literature has characterized alcohol-related reinforcement as proportional to total reinforcement from an individual’s environment [15,16,17,18,19]. Furthermore, given that research has indicated that women tend to have lower proportionate substance-related reinforcement relative to men, researchers often account for gender in their examinations of proportionate alcohol reinforcement and use behavior [23].

Extending behavioral economic theory to alcohol and cannabis co-use, a combination of high valuation of alcohol and limited sources of alcohol-free reinforcement may increase the degree to which young adults (1) use alcohol and (2) use cannabis to amplify the rewarding effects of alcohol (effects that may be highly valued as a source of reinforcement). Preliminary evidence has indicated that young adults who engaged in co-use of alcohol and cannabis had a higher valuation of alcohol [24,25,26], obtained greater proportionate reinforcement from substance-related activities [27], and engaged in fewer substance-free activities [28] compared to individuals who only engaged in alcohol use. For example, in a study with college students, researchers found that individuals with greater proportionate reinforcement from substance-related activities were more likely to engage in co-use. An implication from behavioral economic theory and the existing research may also be that greater proportionate reinforcement from alcohol-free activities may be associated with lower co-use. However, no prior studies have examined this possibility. Thus, the aim of the study was to investigate the association between alcohol-free reinforcement and days of co-use of alcohol and cannabis among a sample of college freshmen. We hypothesized that a higher proportion of reinforcement from alcohol-free activities would be negatively associated with the frequency of alcohol and cannabis co-use (H1).

## 2. Materials and Methods

### 2.1. Participants and Procedures

The current study used baseline data from a pilot study designed to test a behavioral activation intervention administered in a college freshman orientation course [29]. Students were college freshmen who were enrolled in four freshman orientation course sections in the fall of 2019. The students were offered an opportunity to participate in the study during the first week of class. Study procedures were approved by the Institutional Review Board of the University of Kansas, USA. All participants >18 years old provided written informed consent, and those aged 17 provided assent and obtained written parental permission to participate. The baseline visit for the study consisted of surveys assessing substance use behavior, mental health, and sources of reinforcement. Participants were provided extra credit toward their course grade for completing the surveys (or for completing an alternative assignment if they declined participation in the study). All participants (N = 86) who provided informed consent and completed the baseline assessment in the parent study were included in the analysis for the current study.

### 2.2. Measures

#### 2.2.1. Reinforcement from Alcohol-free Activities

Reinforcement from alcohol-free and alcohol-involved activities was measured by the Adolescent Reinforcement Survey Schedule—Alcohol Use Version (ARSS-AUV). In the ARSS-AUV, participants rate the frequency with which they engaged in 45 activities and their level of enjoyment of each activity during the past 30 days [30]. Participants rate each activity twice: once for activities involving alcohol use and again for activities not involving alcohol use. The frequency ratings are on a 5-point Likert scale from 0 (0 times in the past 30 days) to 4 (more than once a day). Enjoyment ratings are on a 5-point Likert scale ranging from 0 (unpleasant) to 4 (extremely pleasant). To obtain a reinforcement score, the cross-product of each activity is calculated by multiplying the frequency and enjoyment ratings for each activity. In addition to characterizing total reinforcement for alcohol-involved activities and alcohol-free activities, the ARSS-AUV has five subscales that can be used to characterize alcohol-involved and alcohol-free reinforcement across five activity areas: peer interaction (14 items; e.g., going to places with friends), family interaction (7 items; e.g., talking with family/siblings), dating (9 items; e.g., going out to eat), sexual activities (4 items; e.g., sexual intercourse), and school activities (3 items; e.g., going to school) [30]. The ARSS-AUV has demonstrated acceptable test-retest reliability and concurrent validity among the college sample [15]. In the current study, there was strong internal consistency for the overall measure (α = 0.93 for alcohol-free and α = 0.97 for alcohol-involved) and for each subscale (peer interaction α = 0.94 for alcohol-free and 0.96 for alcohol-involved; family interaction α = 0.88 for alcohol-free and 0.96 for alcohol-involved; dating α = 0.93 for alcohol-free and 0.96 for alcohol-involved; sexual activities α = 0.83 for alcohol-free and 0.86 for alcohol-involved; and school activities α = 0.82 for alcohol-free and 0.88 for alcohol-involved). The proportion of reinforcement from alcohol-free activities was calculated as the ratio of alcohol-free sources of reinforcement to the total reinforcement (i.e., the sum of alcohol-free and alcohol-involved sources of reinforcement) for use in analysis.

#### 2.2.2. Days of Co-Use of Alcohol and Cannabis

The computerized Time Line Follow Back (TLFB-C), a retrospective calendar-based measure, was used to assess days of co-use of alcohol and cannabis during the past 30 days [31,32]. Participants reported the total number of standard drinks they consumed each day and indicated their cannabis use each day as a binary yes/no. The measure has demonstrated good test-retest reliability and concurrent validity for quantifying alcohol and cannabis use [33,34,35]. Co-use of alcohol and cannabis was identified as the number of days in which participants reported drinking alcohol and using cannabis on the same day, summed over the past 30 days.

### 2.3. Data Analysis

Data analysis was conducted using R Statistical Software [36] with the pscl package (version 1.5.5) [37]. In alignment with the study purpose, zero-inflated regression models were used to characterize the association between alcohol-free reinforcement and days of alcohol and cannabis co-use. The data were first examined to assess their distributional properties and correlations (see Table S2 in the supplemental information section). The outcome variable was days of alcohol and cannabis co-use, which was considered a count variable. The variable was positively skewed and zero-inflated, consistent with the typical distribution of count variables in the literature regarding substance use [38]. Considering the characteristics of the count variable, a zero-inflated Poisson regression (ZIP) model was determined to be the best analytic approach based on recommendations in the literature [38]. The ZIP model was constructed with proportionate alcohol-free reinforcement as the predictor variable and days of co-use as the outcome variable. ZIP regression models consist of two analytic models: one that analyzes count responses (count regression model) and one that predicts zeros in counts (zero-inflated model). ZIP models provide separate estimates for the count regression model in the form of beta estimates and for the zero-inflated model in the form of odds ratios. Applied to the current study model, the zero-inflated model differentiated two groups of individuals who reported zero days of co-use: one group that did not endorse co-use, and the other group that was at risk of engaging in co-use. The count model investigated whether proportionate alcohol-free reinforcement was negatively associated with days of co-use.

In addition to the primary ZIP model, an additional ZIP model was constructed with the mean alcohol-free reinforcement as the independent variable and days of co-use as the dependent variable. The secondary model facilitated an examination of the consistency of the findings across the proportion and mean substance-free reinforcement characterizations.

Finally, because different types of substance-free reinforcement may have different associations with days of co-use, supplemental analyses were conducted with the following subscales specified by the ARSS-AUV: peer interaction, family interaction, dating, sexual activities, and school activities. The findings are summarized in the results and detailed in the supplemental information section.

## 3. Results

### 3.1. Descriptive Statistics

The demographic characteristics of the N = 86 participants are presented in Table 1. Participants were aged from 17 to 19 years old, and females comprised 48% of the sample. Regarding race and ethnicity, most of the sample was white (77%), and 12% of participants were Hispanic. For the full sample, the proportionate alcohol-free reinforcement was 0.73 (SD = 0.17), indicating that on average, participants obtained approximately 73% of their reinforcement from alcohol-free sources of reinforcement. Among individuals who did not engage in co-use in the past 30 days, the proportion of alcohol-free reinforcement was 0.74 (SD = 0.18). Among individuals who engaged in co-use, proportionate alcohol-free reinforcement was 0.68 (SD = 0.11). Approximately 21% of the sample (n = 18) reported engaging in co-use of alcohol and cannabis in the past 30 days. Among individuals who engaged in co-use, the mean number of days of co-use was 4.42 (SD = 4.35).

### 3.2. Zero-Inflated Regression Analysis

Findings from the ZIP analysis are reported in Table 2. Results of the count model for individuals who endorsed co-use indicated that proportionate alcohol-free reinforcement was significantly associated with days of alcohol and cannabis co-use, even when accounting for days of alcohol use and gender (Table 2). The exponentiated coefficients for proportionate alcohol-free reinforcement indicated that for every one unit increase in proportionate alcohol-free reinforcement, the rate of co-use days was 0.962 lower. The results of the zero-inflated model for individuals who reported zero days of co-use indicated that proportionate alcohol-free reinforcement was not a significant predictor of individuals who did not engage in co-use, relative to chance (Table 2). Results of the secondary ZIP model that used mean alcohol-free reinforcement as a predictor variable indicated that alcohol-free reinforcement was significantly associated with lower co-use frequency (Table 3).

Results of the analyses of types of alcohol-free reinforcement indicated that alcohol-free sources of reinforcement from peer interactions, family interactions, and school activities were significantly and negatively associated with days of co-use in the count model (*p* values = 0.004 to <0.001; see supplemental information Table S2), indicating that greater alcohol-free reinforcement from socially oriented and school-based activities was associated with fewer days of co-use among individuals who endorsed co-use.

## 4. Discussion

The current study investigated whether a behavioral economics measure that characterizes reinforcement from alcohol-free activities was associated with alcohol and cannabis co-use among a sample of college freshmen. Findings indicated that among students who engaged in alcohol and cannabis co-use, proportionate alcohol-free reinforcement was significantly associated with days of co-use during the past month, when controlling for days of alcohol use and gender as covariates. Thus, students with greater proportionate reinforcement from alcohol-free activities engaged in fewer days of co-use. Proportionate alcohol-free reinforcement did not meaningfully distinguish individuals who did not report co-use, relative to chance, when controlling for days of alcohol use and gender. Additionally, analyses revealed that alcohol-free reinforcement from socially involved activities (peer and family interactions) and school activities were all associated with lower days of co-use. Altogether, findings identified reinforcement from alcohol-free activities as a factor that may yield lower alcohol and cannabis co-use among individuals who engage in co-use, providing a potential target for future prevention and harm reduction efforts.

Findings from the current study complement the existing literature on reinforcement and substance use broadly, as well as research on reinforcement specifically related to alcohol and cannabis co-use. First, a substantial body of the literature has indicated that overall, greater proportionate reinforcement from substance-related activities may be associated with the risky use of a variety of substances, including alcohol, cannabis, and illicit drugs, among young adult samples [23]. Most of the prior literature has examined associations between proportionate reinforcement and the use of a single substance (e.g., alcohol). However, preliminary evidence regarding reinforcement and co-use of alcohol and cannabis has also indicated similar associations [27,28], supporting the premise that greater reinforcement from substance-involved activities may yield greater substance use behavior. Few studies have examined the potential role of reinforcement from substance-free activities and engagement in substance use, a relationship that is implied in the behavioral economic framework but not often tested. Thus, our findings overall are important in identifying that alcohol-free reinforcement may be associated with less frequent alcohol and cannabis co-use among college freshmen. Notably, our findings were consistent when we examined alcohol-free reinforcement as a proportion of total reinforcement and as a score. The study results are unique as they revealed that reinforcement from one substance (alcohol) may be associated with the co-use of two prevalent substances among college students, thus providing a clear and direct target for potential prevention and harm reduction efforts to address co-use.

Our analyses identified alcohol-free reinforcement from socially involved activities (peer and family interactions) and school activities as being associated with lower days of co-use, suggesting multiple potential target areas for prevention. Limited research has focused on developing interventions to address the co-use of alcohol and cannabis specifically [9], which is a gap in the literature. Our study suggests that one potential intervention approach to address co-use among college students may be to facilitate students’ engaging in more alcohol-free activities in the social and academic areas. This approach would align well with existing behavioral economics-informed intervention approaches, such as brief behavioral activation, which focuses on increasing engagement in reinforcing activities that align with individual goals and values [39,40]. Brief behavioral activation has been used to prevent escalations in alcohol use among college students [41,42,43] and could be adapted to address alcohol and cannabis co-use. However, given that the study had a smaller sample (N = 86), future work with larger samples is needed to replicate our findings before moving into the prevention realm.

The study had several limitations. First, analyses were conducted on cross-sectional data, which precludes causal inferences. In future research, the causal effects of alcohol-free reinforcement on co-use should be investigated. Second, the results were obtained from a small sample collected at a single university. Thus, the results should be replicated by using larger samples. Third, the study did not classify the type of co-use (simultaneous or concurrent) because the measure used did not facilitate this distinction. However, given evidence that both patterns of use may result in greater use-related consequences [4,11,44], our findings are important to consider and identify a potential point of leverage for future prevention efforts. Finally, the sample was comprised of primarily white college freshmen between 17 and 19 years old. Thus, further work is needed to understand the degree to which these findings may generalize to individuals from marginalized racial and ethnic communities, as well as individuals across the age spectrum.

## 5. Conclusions and Implications

The study was the first to examine the association between alcohol-free reinforcement and the frequency of alcohol and cannabis co-use among college freshmen. Among individuals who endorsed co-use, greater proportionate reinforcement from alcohol-free activities was associated with less frequent co-use. If the findings are replicated in larger studies, increasing engagement in alcohol-free sources of reinforcement may be considered a potential target for prevention and harm reduction efforts regarding the co-use of alcohol and cannabis among college students as they enter college.

## Figures and Tables

**Table 1:** Demographic characteristics of participants (N = 86).

Characteristics	Mean (SD) or N (%)
Gender (% Men)	45 (52%)
Age (Years)	18.1 (0.37)
Race	
White	67 (78%)
Black	4 (5%)
Asian or Pacific Islander	7 (8%)
More than one race	5 (6%)
Other	3 (3%)
Ethnicity (% Hispanic/Latino)	10 (12%)

**Table 2:** Association between proportionate alcohol-free reinforcement and days of alcohol and cannabis co-use.

Variables	Estimate ^1^	S.E.	95% CI	*z*	*p* Value
**Count Model**					
Proportion of alcohol-free reinforcement	−3.28	1.35	[−5.93, −0.62]	−2.42	0.016
Days of alcohol use	0.11	0.03	[0.04, 0.17]	3.21	<0.001
Gender [Male]	−0.09	0.32	[−0.72, 0.54]	−0.28	0.777
**Zero-Inflated Model**					
Proportion of alcohol-free reinforcement	−1.68	2.47	[−6.53, 3.17]	−0.68	0.497
Days of alcohol use	−0.21	0.07	[−0.35, −0.08]	−3.08	0.002
Gender [Male]	−0.10	0.66	[−1.39, 1.19]	−0.15	0.881

^1^ For the count model, the estimate was the log-mean; for the zero-inflated model, the estimate was the odds ratio.

**Table 3:** Association between mean alcohol-free reinforcement and days of alcohol and cannabis co-use.

Variables	Estimate ^1^	S.E.	95% CI	*z*	*p* Value
**Count Model**					
Mean alcohol-free reinforcement	−0.21	0.06	[−0.33, −0.10]	−3.69	<0.001
Days of alcohol use	0.14	0.04	[0.07, 0.21]	3.81	<0.001
Gender [Male]	−0.29	0.32	[−0.93, 0.34]	−0.91	0.363
**Zero-Inflated Model**					
Mean alcohol-free reinforcement	0.05	0.16	[−0.26, 0.36]	0.32	0.748
Days of alcohol use	−0.21	0.07	[−0.36, −0.07]	−2.91	0.004
Gender [Male]	−0.19	0.65	[−1.46, 1.07]	−0.30	0.764

^1^ For the count model, the estimate was the log-mean; for the zero-inflated model, the estimate was the odds ratio.

## Data Availability

Not applicable.

## Supplementary Materials

The following supporting information can be downloaded at: https://www.mdpi.com/article/10.3390/ijerph20042884/s1, Table S1: Correlation Matrix for Study Variables; Table S2: Association between Types of Alcohol-Free Reinforcement and Days of Co-use of Alcohol and Cannabis.
