# Supplementary material for: Associations between Alcohol-Free Sources of Reinforcement and the Frequency of Alcohol and Cannabis Co-Use among College Freshmen
Source: Int J Environ Res Public Health. 2023 Feb 7;20(4):2884. doi: 10.3390/ijerph20042884 (PMC9957030; doi:10.3390/ijerph20042884)
Supplement: Supplementary file 1 [file ijerph-20-02884-s001.zip › ijerph-2184003-supplementary.pdf]

Supplemental Information

Pearson correlations for study variables of interested are provided in Table S1. As specified in the main article text, the co-use outcome variable was positively skewed and zero-inflated, thus our characterization of the association between the variables of interest using zero-inflated Poisson regression is a better fit to the data and representation of the relationships among variables given their distributional properties.

Table S1. Correlation Matrix for Study Variables

	1	2	3	4
1	1			
2	-0.07 (p = .530)	1		
3	-0.21 (p = .057)	-0.13 (p = .216)	1	
4	-0.27 (p = .010)	0.11 (p = .313)	0.51 (p < .001)	1

[1] The order of variables are as follows: Proportionate Alcohol-free Reinforcement (1), Mean of Alcohol-free Reinforcement (2), Days of Co-use of Alcohol and Cannabis (3), Days of Alcohol use (4)

Overall, the results of the ZIP models for each alcohol-free reinforcement sub-scale indicated that most types of alcohol-free reinforcement were significantly associated with co-use frequency (Table S2). Specifically, results indicated that among individuals who endorsed co-use, individuals with higher alcohol-free reinforcement from peer interactions, family interactions, and school activities engaged in fewer days of co-use of alcohol and cannabis (Table S2). These associations were significant after controlling for days of alcohol use and gender as covariates. Alcohol-free reinforcement from dating and sexual activities was not significantly associated with days of co-use among individuals who endorsed co-use (Table S2). Alcohol-free reinforcement from the six sub-scales did not meaningfully distinguish individuals who did not engage in co-use, relative to chance (Table S2).

Table S2 Association between Types of Alcohol-Free Reinforcement and Days of Co-use of Alcohol and Cannabis

**Variables**	**Estimate**[1]	**S.E.**	**95% CI**	***z***	***p value***
**Peer Interaction**					
Count model					
Mean Alcohol-Free Reinforcement	-0.21	0.05	[-0.31, -0.11]	-3.99	< .001
Gender [Male]	-0.34	0.31	[-0.93, 0.26]	-1.10	0.273
Days of Alcohol use	0.17	0.04	[0.10, 0.24]	4.87	< .001

**Peer Interaction** *Zero-inflated model*					
Mean Alcohol-Free Reinforcement	-0.01	0.11	[-0.23, 0.20]	-0.11	0.911
Gender [Male]	-0.20	0.66	[-1.48, 1.09]	-0.30	0.764
Days of Alcohol use	-0.20	0.03	[0.07, 0.21]	4.13	< .001
**Family Interaction** *Count model*					
Mean Alcohol-Free Reinforcement	-0.17	0.04	[-0.25, -0.09]	-4.23	< .001
Gender [Male]	0.15	0.34	[-0.52, 0.82]	0.45	0.655
Days of Alcohol use	0.16	0.04	[0.09, 0.24]	4.20	< .001
**Family Interaction** *Zero-inflated model*					
Mean Alcohol-Free Reinforcement	-0.02	0.11	[-0.24, 0.20]	-0.16	0.876
Gender [Male]	-0.07	0.71	[-1.47, 1.32]	-0.10	0.919
Days of Alcohol use	-0.19	0.07	[-0.34, -0.05]	-2.62	0.009
**Dating** *Count model*					
Mean Alcohol-Free Reinforcement	-0.05	0.04	[-0.13, 0.03]	-1.20	0.230
Gender [Male]	-0.55	0.29	[-1.11, 0.01]	-1.92	0.55
Days of Alcohol use	0.11	0.04	[0.05, 0.18]	3.26	< .001
**Dating** *Zero-inflated model*					
Mean Alcohol-Free Reinforcement	0.04	0.08	[-0.11, 0.20]	0.53	0.594
Gender [Male]	-0.25	0.64	[-1.51, 1.00]	-0.40	0.691
Days of Alcohol use	-0.21	0.07	[-0.35, -0.08]	-3.16	0.002
**Sexual Activities** *Count model*					
Mean Alcohol-Free Reinforcement	-0.07	0.04	[-0.14, 0.01]	-1.74	0.083
Gender [Male]	-0.42	0.28	[-0.97, 0.12]	-1.53	0.127
Days of Alcohol use	0.14	0.03	[0.07, 0.21]	4.13	< .001

**Sexual Activities**					
Zero-inflated model					
Mean Alcohol-Free Reinforcement	-0.03	0.09	[-0.21, 0.15]	-0.31	0.757
Gender [Male]	-0.22	0.64	[-1.48, 1.04]	-0.35	0.728
Days of Alcohol use	-0.20	0.07	[-0.34, -0.07]	-2.95	0.003
**School Activities**					
Count model					
Mean Alcohol-Free Reinforcement	-0.09	0.03	[-0.14, -0.03]	-2.87	0.004
Gender [Male]	-0.29	0.29	[-0.86, 0.27]	-1.02	0.308
Days of Alcohol use	0.15	0.03	[0.08, 0.21]	4.27	< .001
**School Activities**					
Zero-inflated model					
Mean Alcohol-Free Reinforcement	-0.003	0.10	[-0.19, 0.18]	-0.04	0.967
Gender [Male]	-0.14	0.65	[-1.42, 1.13]	-0.22	0.824
Days of Alcohol use	-0.20	0.07	[-0.34, -0.07]	-2.92	0.004
